# Attitude to cervical cancer screening and human papillomavirus testing experience in self-sampled Nigerian women

**DOI:** 10.4314/ahs.v24i1.16

**Published:** 2024-03

**Authors:** Ayokunle Moses Olumodeji, Ayodeji Kayode Adefemi, Modupe Olatokunbo Adedeji, Ayokunle Adedayo Ogunyemi, Ifeanyichukwu Augustine Onyeodi, Kabiru Afolarin Rabiu, Olurotimi Ireti Akinola

**Affiliations:** 1 Gynae-oncology unit, Obstetrics and Gynaecology Department, Lagos State University Teaching Hospital, Nigeria; 2 Macedonia Specialist Hospital, Lagos, Nigeria; 3 Obstetrics and Gynaecology Department, Lagos State University College of Medicine, Lagos State University, Nigeria

**Keywords:** Cervical cancer, screening test, cancer screening

## Abstract

**Background:**

Cervical cancer is a disease of major public health significance which can be prevented by adequate screening.

**Objective:**

This study assessed the level of cervical cancer knowledge, attitude to screening and human papillomavirus testing experience in women who self-sampled for cervical cancer screening.

**Methods:**

A descriptive cross-sectional study involving 790 women that had human papilloma virus (HPV) testing at the gynae-oncology unit of the Lagos State University Teaching Hospital. Participants were assessed of their cervical cancer screening knowledge, attitude and HPV testing experience. High risk HPV (hr-HPV) nucleic acid testing was funded by the Clinton Health Access Initiative.

**Results:**

Majority (76.71%) of the respondents exhibited a high level of knowledge of cervical cancer, its causes, risk factors and prevention; and a positive experience with HPV self-sampling reported in 98.1%. hr-HPV positive rate was 13.4%. The most common reason (43%) for not having a cervical screening done was lack of a doctor's request. The most commonly known method of cervical screening by the respondents was Pap Smear test (55.31%).

**Conclusion:**

There is need for more education to improve the level of awareness and uptake of hr-HPV testing for cervical cancer in Lagos. Health care providers are not offering cervical cancer screening enough and this needs to be explored more in future studies.

## Introduction

Cervical cancer is a leading cause of mortality and morbidity in women, especially in sub-Saharan African countries^1^. It is the fourth most common cancer affecting women globally^2^. In Nigeria, cervical cancer is a disease contributing to high levels of cancer deaths^3^. It is preventable if detected and treated early, yet it has a huge burden in Nigeria^4^. Cervical cancer is estimated to affect approximately 500,000 women and 80% of these cases occur in developing countries^5^. In Nigeria, 50.3 million women aged 15 years or older are at risk for developing cervical cancer 6. Current estimates indicate that the number of cervical cancer cases in Nigeria is 14,089 annually, and the number of cervical cancer deaths annually is 8240 7.

One of the most important factors that contribute to the development of cervical cancer is the Human Papilloma Virus (HPV) infection 8. The prevalence of HPV infection in Nigeria is about 24.5% and the incidence of cervical cancer is 250 out of 100,000 women 9. Majority of low/middle-income countries, who bear a large proportion of the disease burden have no universal screening program 10. Although the WHO cervical cancer screening guideline informs practice in Nigeria, the cervical cancer control program in the country is not well-established. Many hospitals and diagnostic laboratories conduct opportunistic screening. Occasionally, both government and non-governmental organizations conduct outreach programs for cervical cancer screening in communities. The absence of a coordinated and comprehensive national screening program is the reason why it is challenging to determine the current level of cervical cancer screening uptake in the country with any degree of certainty.

Another important contributor to the huge burden of cervical cancer in developing countries is the unavailability of accessible cervical screening services^11^ while other factors related to the development of cervical cancer include early sexual exposure, multiple sexual partners and co-infection with HIV 12. Cervical cancer related deaths can be significantly reduced if women have access to adequate screening services 13. However, most women in the rural areas present with advanced disease stages, due to lack of awareness, absence of organized screening programs, and patients delay in seeking health care 13.

Screening helps to identify women with precancerous lesions and treatment could be offered as appropriate 14. Screening is currently viewed as the most effective approach for cervical cancer control, thus leading to reduced incidence and mortality from the disease 15. HPV cervical self-sampling is a screening method recommended by the WHO and hr-HPV DNA testing as an effective approach for the early detection of cervical cancer for women aged 30 years and above 16. Unlike the Pap smear method of cervical screening, HPV testing provides the woman an opportunity to self-collect her sample 17. Findings from current studies suggests that HPV self-sampling is generally associated with increase in the uptake of cervical screening services 18. It is perceived to be highly acceptable to women for its convenience, privacy, cost-effectiveness, comfort and safety 18.

In Nigeria, major obstacles to screening from cultural factors exist. Qualitative researches have emphasized the significant impact of socio-cultural norms, particularly in northern Nigeria, where women often require their husbands' permission for healthcare decisions^19^. Additionally, many women feel uncomfortable being examined by male healthcare workers, and many men prefer female healthcare workers to examine their wives^19^. In such contexts, employing self-sampling could be a more readily accepted approach to cervical cancer screening.

Knowledge of cervical cancer and HPV screening have been found to be consistently low across developing countries and such knowledge poses a challenge to the implementation of cervical cancer programs 20. The knowledge of cervical cancer and early screening have been proven to be the most effective measure for cervical cancer prevention 21. Hence, this study assessed the level of cervical cancer knowledge and HPV screening experience in self-sampled Nigerian women.

## Methods

This was a descriptive cross-sectional study in which 790 consenting women who presented for free cervical cancer screening, using high risk HPV (hr-HPV) DNA testing, at the Gynae-oncology unit of the Lagos State University Teaching Hospital, between 1^st^ March 2022 and 31^st^ of August 2022. They had their socio-demographic information, knowledge of cervical cancer and its prevention, prior attitude to cervical cancer screening and experience of the high-risk HPV testing, obtained using an interviewer-administered semi-structured questionnaire designed for the study. The questionnaire was in English language and its content was validated by expert review and pilot testing with a small sample of participants. Women less than 25years of age and yet to attain sexual debut were excluded from the study.

All eligible women who provided informed consent were provided self-collected sampling kits (Evalyn Brush, Rovers Medical Devices, Oss, Netherlands and Aptima Cervical Specimen Collection Transport Kit, Hologic, Marlborough, Massachusetts, USA). Women who could not self-sample had their sample collected by clinicians. Regarding sample collection, each participant was given the cervical sampling kit. Each contained a pair of disposable gloves, a dry flocked swab, an information leaflet and a 15ml-specimen bottle containing fixative. Convenient and private rooms were provided for self-sample collection. The women were taught verbally by medical doctors to part their labia and gently introduce the flocked swab sampler gently into their vagina until a resistance was felt and then perform a 360-degree rotatory movement with the swab in place. The swab was then removed and its tip broken along a marked line. The broken tip was then placed in the properly labelled specimen bottle containing the fixative and submitted at a designated collection centre from where they were transported within 24 hours to the laboratory for storage and batch analysis. All sampling and testing procedures were funded by the Clinton Health Access Initiative, Nigeria and followed manufacturer's instructions.

Data analysis was mostly descriptive in nature, with categorical variables presented with numerators and percentages and continuous variables. Ethical approval was obtained from the National Health Research and Ethics Committee with protocol number NHREC/01/01/2007.

## Results

### Characteristics of the respondents

A total of 790 Nigerian women were examined. Table 1 shows the characteristics of the study sample of Nigerian women. More than half of the respondents (61.27%) were aged between 30-49. Most participants (80.38%) were married while 12.78% were single. About 53% had attended tertiary education and 26.08% of the respondents had postgraduate education, while only 0.13% had no formal education. Most respondents (89.14%) resided in the urban area while 10.86% resided in the rural area.

**Table 1:** Characteristics of the respondents

Variable	Frequency n=790	Percentage (%)
**Age**		
<30	88	11.14
30-49	484	61.27
50-69	215	27.22
>70	5	0.38
**Marital status**		
Single	101	12.78
Married	635	80.38
**Total number of births**		
0	146	18.48
1-4	586	74.18
>= 5	58	7.34
**HIV Status**		
Negative	726	91.90
Not known	52	6.58
Positive	12	1.52
**Geographical area of residence**		
Urban	681	89.14
Rural	83	10.86
**Contraceptive history**		
Currently being used	49	6.20
No	303	38.25
Not known	7	0.89
Used previously	431	54.56
**Highest level of education**		
No formal education	1	0.13
Primary education	29	3.67
Secondary education	121	15.32
Tertiary education	419	53.04
Postgraduate education	206	26.08

### Knowledge about cervical cancer

As shown in Table 2, we found that 76.71% have heard of cervical cancer, while 21.39% were not aware of cervical cancer. 39.2% of the respondents knew about the major cause of cervical cancer and 74.44% correctly identified Human Papilloma Virus (HPV) as the viral infection which causes cervical cancer. 47.59% of the respondents believed they were not at risk of cervical cancer. In Table 2, the majority of the respondents (67.4%) agreed that cervical cancer can be prevented and almost half of the respondents (49.11%) were aware of the fact that vaccination can help prevent cervical cancer. The most commonly known method of cervical screening among the respondents was Pap smear (55.31%) while the least methods known was HPV testing (26.54%), Visual inspection with Lugol's Iodine (9.08%), and Colposcopy (11.87%). 30.73% of the respondents had never heard of any cervical cancer screening method. There was a generally variable level of awareness of the risk factors for cervical cancer among the respondents with the correctly identified factors being multiple sexual partners (50%), early exposure to sexual relations (29.49%), having a partner with multiple sexual partners (30.33%), and HPV infection (38.73%). 21.90% mentioned Bacteria vaginal infection as a risk factor.

**Table 2:** Knowledge of cervical cancer

Variable	Frequency n=790	Percentage (%)
**Heard of cervical cancer**		
No	169	21.39
Yes	606	76.71
**Are you at risk of cervical cancer**		
No	376	47.79
Yes	90	11.39
**Aware of major cause of cervical cancer**		
No	93	11.77
Yes	313	39.2
**Viral infection which causes cervical cancer**		
HIV	6	1.92
Hepatitis B Virus	1	0.32
Human Papilloma Virus	233	74.44
**Cervical cancer is preventable**		
No	22	2.78
Yes	532	67.34
**A vaccine that prevents cervical cancer**		
No	51	6.46
Yes	388	49.11
**Methods of cervical screening known** *		
Visual Inspection with Lugol's Iodine	65	9.08
Pap Smear	396	55.31
HPV Testing	190	25.54
Colposcopy	85	11.87
None	220	30.73
**Risk factors known to cause cervical cancer** *		
Early exposure to sexual relationship	233	29.49
Having multiple sexual partners	395	50.00
Bacteria vaginal infection	173	21.90
HPV infection	306	38.73
Having a partner with multiple sexual partners	287	36.33
I don't know	131	17.54

* Participants could select more than one response

### Prior screening experience

Reviewing the prior screening experience of the respondents as shown in Table 3, more than half (87.85%) of the respondents have not been invited for a cervical screening, while only 3.92% have been invited. More than half of the respondents (58.11%) have not had their cervical screening done and only 40.0% had done theirs. Among those who had done their cervical screening, 70.47% had done the Pap smear test for cervical screening and only 7.87% did the HPV DNA testing. The most common reason for not having a screening done among the respondents was that there was no doctor's request for it (43.41%).

**Table 3:** Prior screening experience

Variable	Frequency n=790	Percentage (%)
**Past screening invitation**		
No	694	87.85
Yes	31	3.92
**Ever had cervical screening**		
No	369	58.11
Yes	254	40.0
**Cervical cancer screening done in the past**		
HPV DNA Testing	20	7.87
Pap Smear	179	70.47
**Reasons for never having done a screening**		
Could not afford it	80	20.49
Fear of result	40	9.76
No doctor's request	178	43.41
Did not feel I can develop cervical cancer	45	10.98
Privacy concerns	25	6.10
Did not think it's important	84	20.49

### HPV screening and result

More than half of the respondents (81.81%) had a negative test result for hr-HPV testing, 13.41% were positive and only 0.82% of the results were invalid. Of women with positive HPV result, three different HPV types were identified with type 16 having a prevalence of 11.34%, type 18 having 13.40% and type 45 having 8.25%. Other high-risk type was also identified among the respondents (82.65%) (Table 4).

**Table 4:** HPV screening and result

Variable	Frequency n=790	Percentage (%)
**HPV test result**		
Negative	598	81.81
Positive	98	13.41
**Method of sample collection**		
Assisted	44	6.47
Self	589	86.62
**HPV Type identified** *		
Type 16	11	11.22
Type 18	13	13.27
Type 45	8	8.16
Other high-risk types	81	82.65
**Satisfied and willing to recommend screening to others**		
No	8	1.9
Yes	418	98.1

* Some participants had more than one hr-HPV type and denominator is the number of HPV positive women

The majority (86.62%) of the samples was self-collected and 6.47% were assisted collections. Most respondents (98.1%) were satisfied with the modality of screening and willing to recommend the screening to others.

## Discussions

This study examined the knowledge of cervical cancer and HPV screening experience with self-sampling among Nigerian women. Overall findings from this study showed that there is a relatively high level of knowledge of cervical cancer among our respondents and a positive experience with HPV self-sampling screening. Majority of the respondents were married (80.38%) and these respondents demonstrated a high level of awareness of cervical cancer (76.71%). The high level of awareness was probably due to the fact that a majority of the respondents were educated, with 53.04% having tertiary education and having Postgraduate education (26.08%). This could partly be the reason more than half of the respondents (55.31%) were aware of Pap smear screening method. However, in contrast to these, findings from similar studies carried out in Lagos and Ogbomoso, Nigeria, showed a relatively low level of knowledge of 12.8% and 22.6% respectively 15, 22. According to previous studies, high level of education is known to be associated with better access to health information 22. Majority of the respondents (55.31%) were aware of Pap smear as one of the screening methods for cervical cancer. This corroborates with findings from a similar study by Oche MO et al., in Sokoto, Nigeria 12. The high level of awareness of cervical cancer and Pap smear demonstrated by the respondents indicated a proper utilization of the screening method. Less than half (25.54%) of the respondents were aware of HPV testing as a screening method for cervical cancer and 7.87% of the respondents had done HPV testing in the past. This indicates a low level of awareness of HPV testing as the screening modality for cervical cancer. HPV testing is a valued and reliable method of testing due to its superior sensitivity and advantages that could result from using self-collected samples 23. Thus, there is a need to raise awareness about HPV testing, in addition to knowledge about cervical cancer and systems should be equipped for HPV testing.

Only about 49% of the study population acknowledged the existence of a vaccine that could protect against cervical cancer. The most effective preventive method of cervical cancer among adolescent girls and other women prior to sexual exposure is the primary prevention by HPV vaccination 7. Therefore, HPV vaccination is highly recommended for the prevention of cervical cancer. Our findings suggest that there is much to be done in this region on increasing awareness of HPV vaccination.

The most common reason for not having a cervical screening done among the respondents was that there was no doctor's request (43.41%) for the screening. Despite the high level of knowledge of the connection between cervical cancer and sexual activity, as well as sexually transmitted diseases, a large proportion (47.59%) of the study subjects believed they were not at risk of cervical cancer. A similar study also found that 34.4% of their respondents felt they were not at risk of the disease 24. Other reasons given for not having a cervical screening done include fear of the result, privacy concerns, unable to afford it, and feeling that it isn't important. The rate of past invitation to screening was found to be very low (3.92%) which indicates a low rate of screening uptake. Government and non-governmental organizations should look into measures to invite women for cervical cancer screening as a means to foster uptake of screening tests.

The rate of HIV status known and contraceptive use was considerably low (6.58% and 38.35% respectively) among the respondents and women who aren't aware of their HIV status and contraceptive use may be opportune to take advantage of cervical screening as it may provide them with opportunities for counselling on HIV testing and contraceptive use.

Findings from our study showed that majority of our respondents (74.44%) correctly identified HPV infection as the primary cause of cervical cancer. This is supported by similar studies conducted by Oche MO et al., and Olubodun T. *et al.*, 12, 22.

The rate of hr-HPV positivity found from the respondents was 13.41% and this is higher than the positivity rate (3.5%) of HPV detected from a similar study 25. The low HPV positivity rate (13.41%) facilitated the use of HPV test as the primary testing for cervical cancer and this is relevant for other countries with low HPV prevalence rate. Different types of HPV types including HPV 16 (11.34%), HPV 18 (13.40%), and HPV 45 (8.25%) were identified in the respondents screening results. Other high-risk types were identified with a seroprevalence of more than 82.7%. According to previous studies, 95% of cervical cancer was found to be related to high-risk HPVs 26.

The experience with HPV screening among the respondents was very good as 98.1% of the respondents were satisfied with the modality of screening and willing to recommend it to others. Majority (86.62%) of the study samples was self-collected and this made it more convenient for the respondents. However, in a similar study conducted in Nigeria, it was reported that the respondents (12.8%) had a fair experience with self-sampling. Findings from our study indicated that self-sampling was largely a success in the women studied and may be acceptable in the Nigerian population. A limitation of this study is that specific questions to further explore the women's experience of self-sampling were not asked. The high satisfaction rate in this study suggests that, overall, the self-sampling modality is likely to be more acceptable to Nigerian women due to its alignment with cultural norms,^19^ increased privacy, and reduced discomfort associated with the screening process.^27^ It has the potential to increase cervical cancer screening rates and ultimately contribute to better women's health outcomes in Nigeria. Self-sampling has been recommended by the WHO as a primary HPV based screening and as an approach to cervical cancer prevention 27. Previous studies also recommend health literacy including knowledge about the disease and early screening as an effective measure of cervical cancer prevention 28.

## Conclusion

The women in this study demonstrated a high level of knowledge of cervical cancer, its cause, risk factors and prevention. In addition, the respondents experience with HPV self-sampling screening was positive. Although there is a good knowledge and positive experience towards cervical cancer and screening, still there is a need for education on cervical cancer and how to improve the awareness of cervical cancer among girls and older women in Lagos. Health care providers are not offering cervical cancer screening enough and this major hurdle for screening needs to be explored more in future studies. For a start, despite busy clinics, medical providers must routinely offer and provide cervical cancer screening services to eligible clients. In addition, government and non-governmental organizations should further develop measures to make use of educational intervention to raise awareness on cervical cancer knowledge, HPV testing and self-sampling.
